# Gel polymer electrolyte-based dual screen-printed electrodes for the headspace quantification of 4-ethylphenol and ethanethiol simultaneously in wines

**DOI:** 10.1007/s00604-024-06220-8

**Published:** 2024-03-19

**Authors:** Paula Portugal-Gómez, Olga Domínguez-Renedo, M. Asunción Alonso-Lomillo

**Affiliations:** Analytical Chemistry Department, Faculty of Sciences, University of Burgos, Pza. Misael Bañuelos S/N, 09001 Burgos, Spain

**Keywords:** 4-Ethylphenol, Ethanethiol, Wine analysis, Dual screen-printed carbon electrodes, Gas sensor, Gas-phase amperometry, Room temperature ionic liquid

## Abstract

The identification and correction of negative factors, such as 4-ethylphenol and ethanethiol, is important to comply with food safety regulations and avoid economic losses to wineries. A simple amperometric measurement procedure that facilitates the simultaneous quantification of both compounds in the gas phase has been developed using fullerene and cobalt (II) phthalocyanine-modified dual screen-printed electrodes coated with a room temperature ionic liquid-based gel polymer electrolyte. The replacement of the typical aqueous supporting electrolyte by low-volatility ones improves both operational and storage lifetime. Under the optimum conditions of the experimental variables, Britton Robinson buffer pH 5 and applied potentials of + 0.86 V and + 0.40 V for each working electrode (vs. Ag ref. electrode), reproducibility values of 7.6% (*n* = 3) for 4-ethylphenol and 6.6% (*n* = 3) for ethanethiol were obtained, as well as capability of detection values of 23.8 μg/L and decision limits of 1.3 μg/L and 9.2 μg/L (α = β = 0.05), respectively. These dual electrochemical devices have successfully been applied to the headspace detection of both compounds in white and red wines, showing their potential to be routinely used for rapid analysis control in wineries.

**Graphical Abstract:**

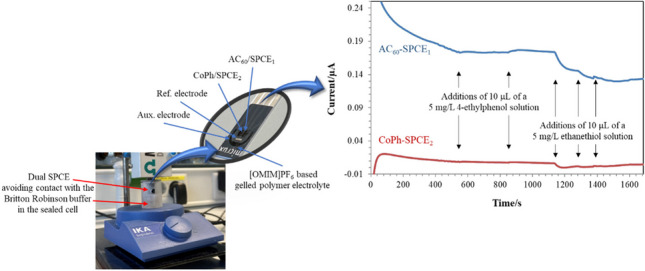

**Supplementary Information:**

The online version contains supplementary material available at 10.1007/s00604-024-06220-8.

## Introduction

The production of high-quality wines that do not contain any faults or defects is a priority for winemakers. In addition, to comply with food safety regulations, the identification and correction of negative factors, such as off-odors, can avoid economic losses, since pleasure on consumption and reputation strongly influence the image of a wine and its purchase. Among them, volatile phenols, such as 4-ethylphenol reminiscent of stables and sweaty saddles, and volatile sulfur compounds, such as ethanethiol reminiscent of rotten-onion at threshold levels and fecal odor at higher levels, clearly have a negative influence on a wine quality [1, 2]. On the one hand, contaminated wines by *Brettanomyces* yeast can suffer the enzymatic transformation of grape hydroxycinnamic acids into ethylphenols [3, 4]. On the other hand, the formation of traces of mercaptans can be considered only a small anomaly in the biochemistry of fermentation, but their sensory impact can even alter the aroma of bottled wines [5, 6]. Thus, simple measurement procedures that facilitate the simultaneous quantification of these notable and harmful compounds are necessary in wine quality control, with the aim of making early preventive diagnoses. In addition to chromatographic procedures [7–14], analytical instrumentation based on the use of electrochemical sensors has also been developed to detect electroactive redox species in a matrix as complex as wine [15]. The latter stand out for their high sensitivity, low cost, and, above all, for their compact size given their ability to be miniaturized, which makes them ideal portable instruments for in situ measurements. In this way, the voltammetric currents of 4-ethylphenol and ethanethiol, representing mercaptans, have been registered at hanging mercury drop electrodes [6], graphite epoxy composites [16], molecularly imprinted polypyrrole-based glassy carbon electrodes [17, 18] or screen-printed carbon electrodes (SPCEs) modified by gold nanoparticles [19], fullerene (C_60_) [20, 21], and cobalt (II) phthalocyanine (CoPh) [22]. Electrochemical gas sensors, which are based on the detection of electron transferred during the oxidation or reduction of volatile targets at the working electrode, are quite attractive for this aim since the number of species that can interfere the analytical signal is reduced. Moreover, these sensors hold improving practices in wine analysis given that the sample is not altered [20, 22]. Activated C_60_-modified SPCEs (AC_60_/SPCEs) and CoPh-modified SPCEs (CoPh/SPCEs) have already highlighted their ability to individually quantify 4-ethylphenol and ethanethiol in wine samples, using a two-step procedure in which the voltammetric measurements are carried out in solution after an incubation process in gas phase [20] or directly at the headspace of a sealed cell [22]. The main drawbacks of these sensors are related to their operational and storage lifetime, limited due to the evaporation of the nonreactive electrolyte in which the electrodes must be immersed (supporting electrolyte), which were pre-loaded by adsorption at the devices. As it has been described at the bibliography, the typical aqueous supporting electrolyte can be replaced by low-volatility materials, such as Nafion [23] or room temperature ionic liquids (RTILs) [24, 25]. Up to now, diverse approaches have been attempted for the implementation of solid supporting electrolytes onto electrochemical devices, ranging from the use of direct Nafion [23], RTIL [26] or RTIL/ethanol mixtures [27] drop-casted onto the sensor surface, RTILs dried at 60 °C for 1 day [28], to blends of RTILs, poly(vinylidene fluoride) (PVDF), and organic solvents such as 1-methyl-2-pyrrolidone (NMP) [29] or N,N-dimethylformamyde (DMF) [30, 31]. In this work, the performance of the simultaneous headspace amperometric quantification of 4-ethylphenol and ethanethiol, associated to important organoleptic defects, has been studied using AC_60_ and CoPh-modified dual SPCEs coated with different solid-state supporting electrolytes. These dual electrochemical devices have successfully been applied to the headspace detection of both compounds in white and red wines, showing their potential to be routinely used for rapid analysis control in wineries, that is, as a kind of point-of-care application.

## Experimental

### Chemicals and instrumentation

All chemicals were used of analytical reagent grade. Solutions were prepared in Milli-Q water (Millipore, Bedford, MA, USA). Britton Robinson buffer, 0.04 M phosphoric acid (Panreac, Barcelona, Spain), acetic acid 0.04 M (VWR Chemical, Fontenay, France) and boric acid 0.04 M (Panreac, Barcelona, Spain), and 0.1 M potassium chloride (Merck, Darmstadt, Germany) solutions were used. A 1 M NaOH solution (Ecros, Barcelona, Spain) was used to adjust the pH.

Standard solutions of 4-ethylphenol (Alfa Aesar, Haverhill, Massachusetts, USA) and ethanethiol (VWR Chemicals, Rosny-sous-Bois, France) were prepared by dissolving the adequate amount of each reagent in Milli-Q water.

C_60_ (Acros Organics, Geel, Belgium) and CoPh (Alfa Aesar, Karlsruhe, Germany) solutions were prepared in dichloromethane (Panreac, Barcelona, Spain) and ethanol (VWR Chemicals, Rosny-sous-Bois, France), respectively. 1.0 M potassium hydroxide solutions (Carlo Erba, Val de Reuil, France) were used to activate C_60_.

Nafion (Sigma-Aldrich, Steinheim am Albuch, Germany), 1-methyl-3-octylimidazolium hexafluorophosphate ([OMIM]PF_6_, Sigma-Aldrich, San Luis, MO, USA), 1-n-butyl-3-methylimidazolium hexafluorophosphate ([BMIN]PF_6_, Thermo Fisher Scientific, Waltham, MA, USA), PVDF (Thermo Fisher Scientific, Waltham, MA, USA), and NMP (VWR International, Radnor, Pennsylvania, USA) were used as solid supporting electrolytes.

A potentiostat PalmSens4 (PalmSens, BV, Houten, The Netherlands) and SPCEs based on a 3-electrode configuration (DRP-C11L, Metrohm DropSens, Oviedo, Spain) and dual SPCEs (ED-D2PE-C, MicruX Technologies, Gijón, Spain) contain a silver reference, a carbon auxiliary and 2-carbon working electrodes were used for the electrochemical measurements. All the working potentials in this work were applied vs this reference electrode.

### Dual SPCE modification

**Fig. 1:**
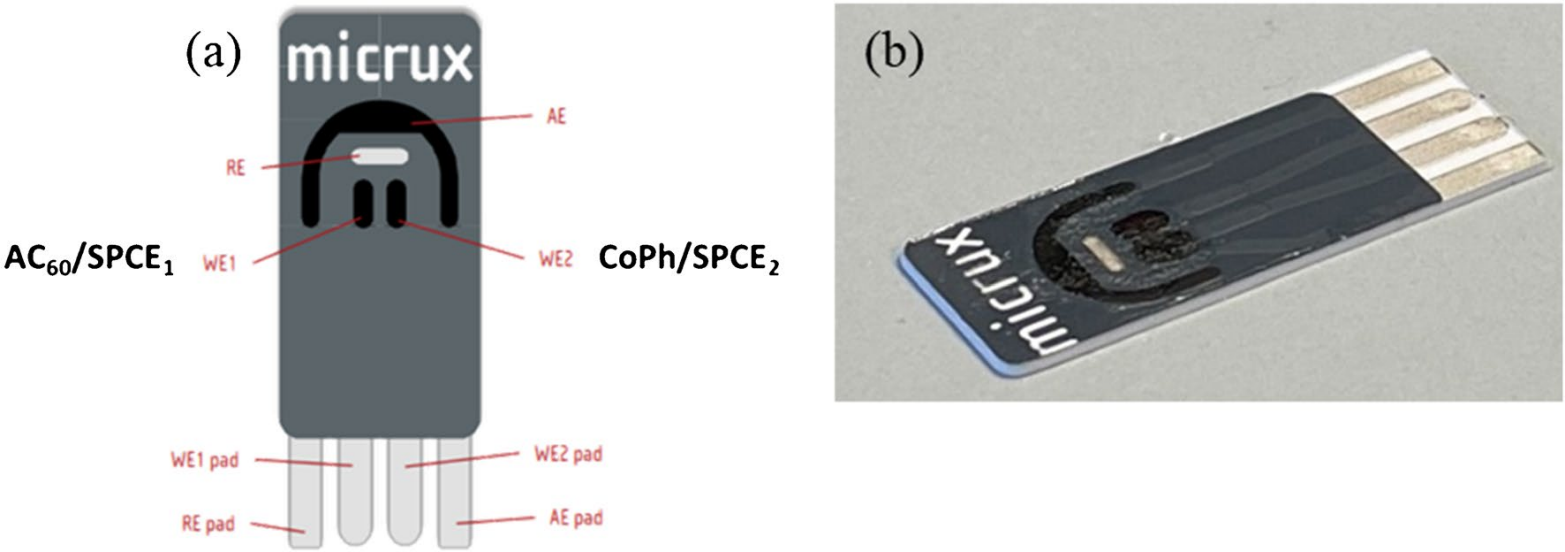
**a** AC_60_ and CoPh-modified dual SPCEs and **b** AC_60_ and CoPh-modified dual SPCEs, coated with a gel polymer electrolyte based on the use of [OMIM]PF_6_, for the simultaneous detection of 4-ethylphenol and ethanethiol

The 2-working electrodes were individually modified using C_60_ and CoPh in order to build a sensitive and selective sensor for both 4-ethylphenol and ethanethiol, one-to-one, according to previously described procedures (Fig. 1a) [20, 22]. Forty microliters of a 0.1 mg/mL solution of C_60_ in dichloromethane was coated on the carbon working electrode surface and allowed to dry at room temperature. It has been reported a low electrochemical activity of C_60_ in aqueous solution, so an activation step was carried out in order to enhance its electron transfer capacity to the electrode surface. Fifty microliters of a 1 M KOH solution was dropped onto the device and a cyclic voltammogram between 0 and − 1.5 V was recorded at 10 mV/s. The partially reduced C_60_-modified electrode (AC_60_/SPCE_1_) becomes conducting in this way [20, 32]. The second working electrode was modified by drop-casting a volume of 10 μL of a 5% w/v solution of CoPh in ethanol and left to dry at room temperature (CoPh/SPCE_2_) [22].

### AC_60_ and CoPh-modified dual SPCEs coated with gel polymer electrolytes based on the use of RTILs

The four electrodes must be immersed in supporting electrolyte to make up the electrochemical cell completely. The gel polymer electrolyte was prepared by dispersing PVDF in NMP at a mass ratio of 1:10 and stirred at 500 rpm for 10 min at 60 °C. Then, this mixture was added to [OMIM]PF_6_ or [BMIN]PF_6_ in a mass ratio of 1:2 and stirred until homogenization for a few minutes at 60 °C [33]. Finally, 3 μL of the gel mixture was deposited onto the four electrodes, forming a thin and reproducible film. The developed devices were kept at room temperature until use (Fig. 1b).

### Electrochemical measurements

Modified dual SPCEs were placed on top of a sealed cell containing 1 mL of Britton Robinson buffer pH 5, except for the optimization process, avoiding contact. The cell was manufactured using a UV resin for 3D printers with a side hole that allows the introduction of samples (Figure S1). Amperometric measurements were performed by applying a potential of + 0.86 V to AC_60_/SPCE_1_ and + 0.40 V to CoPh/SPCE_2_, except for the optimization process. Once constant intensities were recorded, a volume of 50 μL of a 0.5 mg/L solution of both ethanethiol and 4-ethylphenol was added to the cell, recording the corresponding increase in current due to the oxidation process that takes place on the surface of both electrodes.

Commercial wine samples, analyzed without any kind of sample pre-treatment, and fortified wine samples at 23.8 μg/L or 43.6 μg/L of 4-ethylphenol and ethanethiol, prepared by adding the corresponding amount of solutions of each analyte to the wine sample, were analyzed as well by the standard addition method [34]. Thus, once constant intensities were recorded, a volume of 50 μL of the corresponding wine sample (unknown sample) was first added to the electrochemical cell, following by additions of a solution of known concentration of both ethanethiol and 4-ethylphenol.

## Results

The simultaneous headspace amperometric quantification of 4-ethylphenol and ethanethiol was attempted using modified dual SPCEs, containing AC_60_/SPCE_1_ and CoPh/SPCE_2_ as it has been described above. Careful choice of the composition of the supporting electrolyte should improve the performance of this device in terms of operational and storage stability, so low-volatility materials were studied to replace the typical aqueous supporting electrolyte.

The behavior of AC_60_ and CoPh-modified dual SPCEs covered with Nafion was studied [23]. A volume of 5 μL of a mixture of a Nafion solution (2% in ethanol) and buffer, in different percentages (100:0, 50:50, and 25:75), was dropped onto the modified dual SPCEs and left to dry at room temperature. The obtained results were similar in all the three experiences: while nice signals for ethanethiol were obtained, no oxidation currents were registered for 4-ethylphenol due to a kind of blocked effect of the AC_60_/SPCE_1_ by the Nafion membrane.

As an alternative, RTILs can also be used as electrolytes on solid supports considering their non-volatility and the high ionic conductivity [24]. So, RTILs such as [OMIM]PF_6_ and [BMIM]PF_6_ were directly deposited by drop-casting onto the modified dual devices, but no reproducible currents were recorded due to their leakage. Consequently, the RTIL-modified devices were left to dry at 100 °C for 1 h and washed to remove the excess of electrolyte prior to use. In the case of AC_60_ and CoPh-modified SPCEs covered with [OMIM]PF_6_, the most influential experimental variables, pH of the supporting electrolyte and applied potential, were individually optimized to obtain the highest current for the detection of 4-ethylphenol and ethanethiol.

2^2^ central composite designs were carried out taking as response to the oxidation current of 9.8 µg/L solutions of both analytes. The analysis of the variance of the obtained data, performed using the Statgraphics software [35], showed that the values that maximize the oxidation response of 4-ethylphenol were + 0.76 V and pH 2.8, and + 0.55 V and pH 5.1 in the case of ethanethiol. However, poor results were obtained either at pH 2.8 or at 5.1 when AC_60_ and CoPh-modified dual SPCEs covered with [OMIM][PF6] or [BMIM]PF6 were used, since the response was similar in both electrodes although a different potential was applied to each one. This was attributed to the fact that a mixture of both modifications was possibly produced when covering the electrodes with the RTILs.

Thus, solid polymer electrolytes based on the use of both RTILs were also attempted. Membranes were prepared by combining different ratios of a RTIL, PVDF as polymer matrix and NMP as solvent (Table S1), stirring them until a homogeneous mixture was formed and left to dry at 100 °C during 60 min [29]. It was observed that the greater the amount of polymer used in the mixture, the worse the adhesion of the membrane to the device. On many occasions, this membrane even ended up falling into the solution. In the cases where the membrane was well adhered to the surface, no quantifiable oxidation signals for both analytes were recorded, which was attributed to a scarce diffusion of the volatile analytes to the working electrodes (Figure S2). It has already been reported that the thermal treatment of PVDF-based solid polymer electrolytes influences the sensitivity, since higher crystallization temperature resulted in lower porosity [29, 33].

**Fig. 2:**
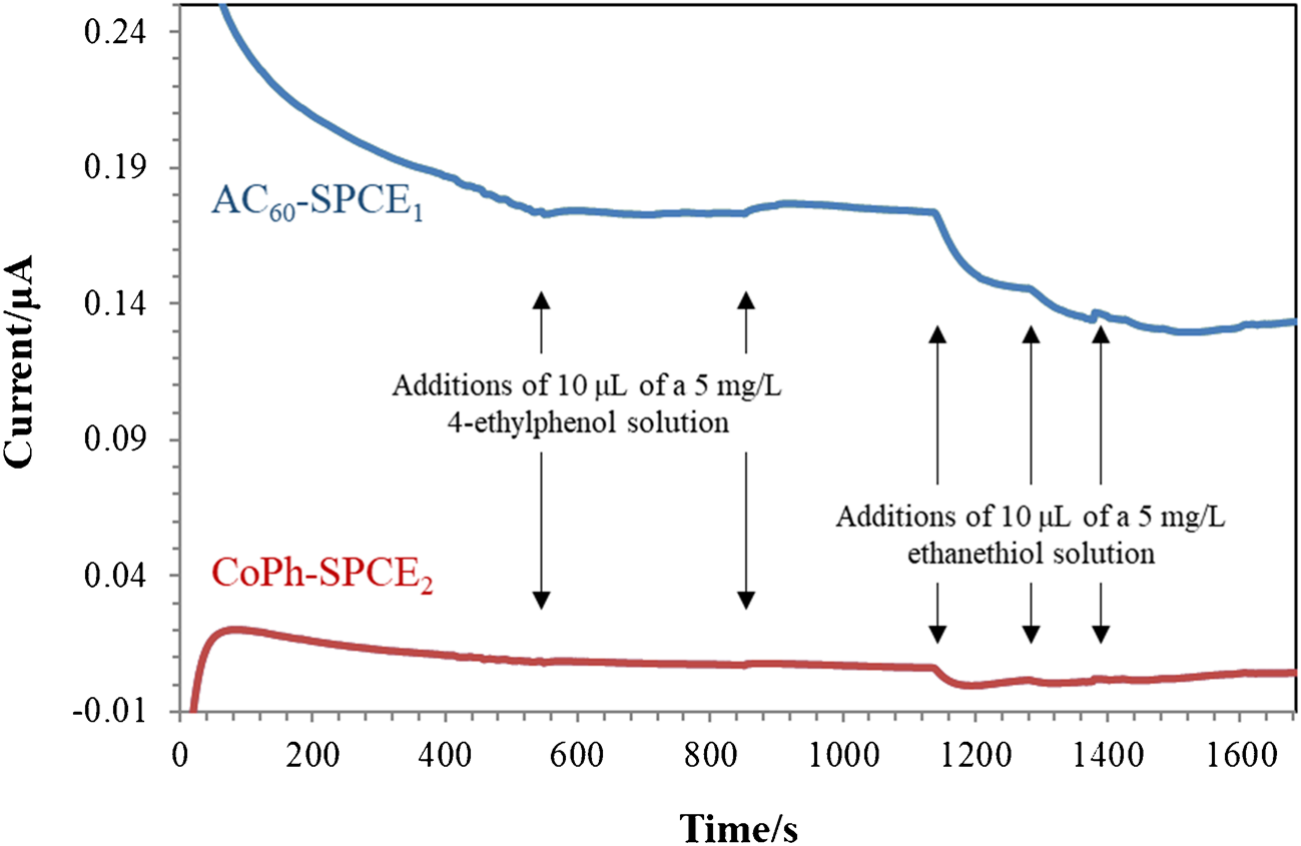
Headspace amperometric measurements performed for the detection of 4-ethylphenol and ethanethiol in Britton Robinson buffer pH 5 using a dual AC_60_/SPCE_1_ (applied potential, + 0.86 V) and CoPh/SPCE_2_ (applied potential, + 0.40 V) device modified by a gel polymer electrolyte based on [OMIM]PF_6_

So, a gel polymer electrolyte was built using a lower PVDF concentration and a lower drying temperature, according to the procedure described in “AC_60_ and CoPh-modified dual SPCEs coated with gel polymer electrolytes based on the use of RTILs” section, reaching in this way both nice membrane adhesion and oxidation currents for both 4-ethylphenol and ethanethiol when [OMIM]PF_6_ was used. In order to obtain the best signal for the simultaneous detection of both analytes, different pH values in the range from 2 to 5 and applied potentials from + 0.1 to + 1.0 V were studied. The optimum values that led to the maximum oxidation currents were Britton Robinson buffer pH 5 and applied potential of + 0.86 V for AC_60_-SPCE_1_ and + 0.4 V for CoPh-SPCE_2_ (Fig. 2). Under these conditions, increasing oxidation currents were registered with increasing concentrations of ethanethiol at the CoPh-SPCE_2_, while the AC_60_-SPCE_1_ was only able to record the effect of the addition of the solution into the electrochemical cell. On the contrary, increasing oxidation currents were registered with increasing concentrations of 4-ethylphenol at the AC_60_-SPCE_1_, but no signals were recorded at the CoPh-SPCE_2_.

**Fig. 3:**
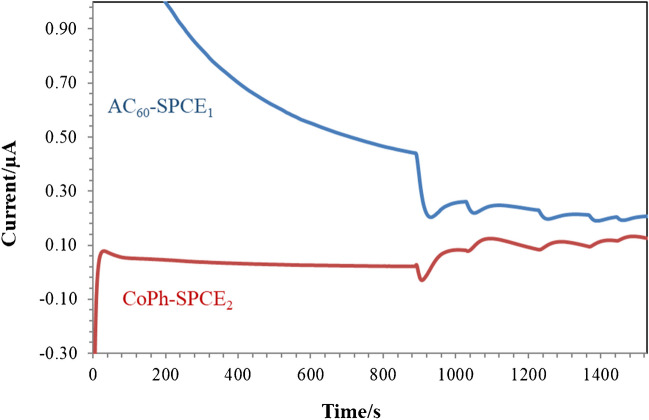
Headspace amperometric measurements performed for the simultaneous detection of 4-ethylphenol and ethanethiol in Britton Robinson buffer pH 5 using a dual AC_60_/SPCE_1_ (applied potential, + 0.86 V) and CoPh/SPCE_2_ (applied potential, + 0.40 V) device modified by a gel polymer electrolyte based on [OMIM]PF_6_. Each addition corresponds to 50 μL of a 500 μg/L of 4-ethylphenol and ethanethiol solution

Different calibration curves were constructed to study the performance of this procedure in terms of precision and capability of detection, under these optimum conditions, by adding both 4-ethylphenol and ethanethiol simultaneously into the electrochemical cell, in such a way that their concentration ranges from 23.8 to 83.3 μg/L (Fig. 3). Outliers’ points with a studentized residual above 2.5 in absolute value were removed to provide a proper evaluation of the calibration parameters obtained by ordinary least squares regression [35]. In the case of the reproducibility, the slopes of calibration curves recorded using different AC_60_ and CoPh-modified dual SPCEs coated with a [OMIM]PF_6_-based gel polymer electrolyte were evaluated in terms of relative standard deviation (RSD), reaching a value of 7.6% (*n* = 3) for the AC_60_/SPCE_1_ and 6.6% (*n* = 3) for the CoPh/SPCE_2_ (Table 1 and figure S3). When these kinds of measurements were carried out using a single AC_60_ and CoPh-modified dual SPCEs coated with a [OMIM]PF_6_-based gel polymer electrolyte, it already observed a decrease in the slope of the third calibration curve (Table S2 and figure S4). Although the repeatability of these devices was slightly worse than their reproducibility, this was not considered a major drawback considering their disposable nature and ease of construction. Decision limit (CCα) and capability of detection (CCβ) were also estimated on the base of the validated calibration curves, according to the ISO 11843 approach [36], using the DETARCHI program [37]. CCα of the procedure is defined as the lowest concentration level at which the method can discriminate if the analyte of interest is in the sample with a probability of 1–α, where α is the probability of false positive. In this way, limit of decisions of 1.3 μg/L and 9.2 μg/L were achieved for 4-ethylphenol and ethanethiol, respectively. Likewise, CCβ is estimated as the lowest concentration level of analyte that the method can detect with a probability of 1–β, where β is the probability of false negative. When using α = β = 0.05 values, both results were under the concentration of the first standard, 23.8 μg/L, so this value was taken as the capability of detection for both analytes from an analytical point of view [38]. This performance is as good as that achieved using individual electrochemical gas sensors (Table S3), with the great advantage that the sensors developed in this work allow the simultaneous detection of both analytes and their use for a longer time, due to the use of gel polymer electrolytes. In fact, it was possible to use these devices at least 15 days after their manufacture, having been stored at room temperature.

**Table 1:** Calibration parameters obtained through ordinary least squares regressions without outliers for the determination of 4-ethylphenol and ethanethiol using different AC_60_ and CoPh-modified dual SPCEs coated with a [OMIM]PF_6_-based gel polymer electrolyte under the optimum conditions

4-Ethylphenol concentration range (μg/L)	Intercept (nA)	Slope (nA (μg/L)^−1^)	Coefficient of determination (*R*^2^)	Standard error of estimate (S_yx_)
23.8–83.3	− 1.65	0.14	0.998	0.194
23.8–83.3	− 1.20	0.12	0.998	0.175
23.8–83.3	− 2.30	0.14	0.999	0.036
Ethanethiol concentration range (μg/L)	Intercept (nA)	Slope (nA (μg/L)^−1^)	Coefficient of determination (*R*^2^)	Standard error of estimate (S_yx_)
23.8–83.3	1.67	0.13	0.999	0.136
23.8–83.3	− 0.64	0.15	0.999	0.126
23.8–83.3	0.96	0.13	0.994	0.246

The selectivity of the developed method was studied, considering the possibility that other volatile phenols and thiols present in the wine matrix could alter the amperometric response of 4-ethylphenol and ethanethiol. For this purpose, two volatile phenols were selected: 4-ethylguaiacol, which has a structure like 4-ethylphenol and 4-vinylphenol, a precursor of 4-ethylphenol; and a thiol, 4-mercaptobenzoic acid. Under the optimum measurements’ conditions for the simultaneous determination of 4-ethylphenol and ethanethiol, no alteration in the amperometric response was obtained due to the presence of these compounds, so they were not considered interferents.

The applicability of the method was verified by the simultaneous determination of 4-ethylphenol and ethanethiol in different wine samples. Four different commercial samples from different grape variety were studied without finding the presence of analyte in any of them. Recovery experiments were also performed by the analysis of fortified wine samples at two levels by the standard addition method (Table 2), obtaining values from 91 to 105%, which highlights the applicability of the developed analytical method to be routinely used for rapid analysis control in wineries [34].

**Table 2:** Determination of 4-ethylphenol and ethanethiol in different wine samples using AC_60_ and CoPh-modified dual SPCEs coated with a [OMIM]PF_6_-based gel polymer electrolyte

Sample	Analyte	Concentration added (μg/L)	Concentration found (μg/L)	Recovery (%)
White wine 1	4-Ethylphenol	23.8	22.3 ± 1.7	93.7
	Ethanethiol	23.8	22.8 ± 2.0	95.6
	4-Ethylphenol	46.7	44.8 ± 2.2	96.0
	Ethanethiol	46.7	43.0 ± 3.8	92.1
White wine 2	4-Ethylphenol	23.8	22.0 ± 2.0	92.4
	Ethanethiol	23.8	21.8 ± 2.8	91.6
	4-Ethylphenol	46.7	46.8 ± 2.4	100.2
	Ethanethiol	46.7	47.8 ± 3.4	102.4
Red wine 1	4-Ethylphenol	23.8	23.92 ± 1.6	100.4
	Ethanethiol	23.8	23.08 ± 1.7	96.9
	4-Ethylphenol	46.7	44.3 ± 3.3	94.9
	Ethanethiol	46.7	42.6 ± 4.4	91.2
Red wine 2	4-Ethylphenol	23.8	24.5 ± 1.1	102.9
	Ethanethiol	23.8	24.9 ± 3.3	104.6
	4-Ethylphenol	46.7	46.0 ± 3.3	98.5
	Ethanethiol	46.7	46.2 ± 3.0	99.0

## Conclusions

AC_60_ and CoPh-modified dual SPCEs coated with a [OMIM]PF_6_-based gel polymer electrolyte enable the simultaneous quantification of 4-ethylphenol and ethanethiol in the gas phase. The use of this gel polymer electrolyte to immerse the four electrodes instead of the typical aqueous ones improves the performance of this device in terms of operational and storage lifetime. In this way, it has been possible to build reproducibility sensors, with RSD of 7.6% (*n* = 3) for 4-ethylphenol and 6.6% (*n* = 3) for ethanethiol, and to use them at least 15 days after their manufacture, having been stored at room temperature, for the quantification of 4-ethylphenol and ethanethiol in white and red wines, obtaining recovery values greater than 91% for both analytes. Headspace amperometric measurements of these notable and harmful compounds, associated to important organoleptic defects in wine, are quite attractive since not only the number of species that can interfere the analytical signal is reduced, but also wine samples are not altered. Considering this successful application, these dual amperometric sensors are promising candidates for use in rapid analysis control in wineries, with the aim of making early preventive diagnoses routinely.

### Supplementary Information

Below is the link to the electronic supplementary material.

Supplementary file1 (DOCX 1344 KB)

## Data Availability

Data available upon request.
