# Supplementary material for: Gel polymer electrolyte-based dual screen-printed electrodes for the headspace quantification of 4-ethylphenol and ethanethiol simultaneously in wines
Source: Mikrochim Acta. 2024 Mar 19;191(4):208. doi: 10.1007/s00604-024-06220-8 (PMC10948468; doi:10.1007/s00604-024-06220-8)
Supplement: Supplementary file 1 — Supplementary file1 (DOCX 1344 KB) [file 604_2024_6220_MOESM1_ESM.docx]

**SUPPLEMENTARY MATERIAL**

**Gel polymer electrolyte-based dual screen-printed electrodes for the headspace quantification of 4-ethylphenol and ethanethiol simultaneously in wines**

**Paula Portugal-Gómez, Olga Domínguez-Renedo and M. Asunción Alonso-Lomillo***

*Analytical Chemistry Department, Faculty of Sciences, University of Burgos, Pza. Misael Bañuelos s/n, 09001 Burgos, Spain.*

*Email: malomillo@ubu.es*


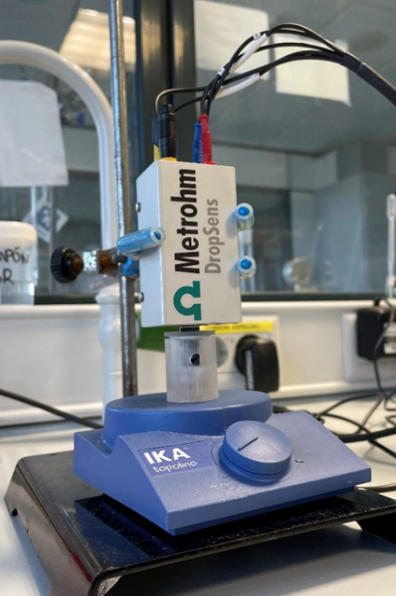


***Figure S1.-*** *Electrochemical cell used for the headspace amperometric measurements performed for the detection of mercaptans and 4-ethylphenol.*

***Table S1.-*** *Amounts of RTIL, PVDF and NMP (w/w/w) used in the mixture to build the solid polymer electrolyte and results obtained in terms of membrane formation and electrochemical measurements.*

|  | **RTIL** | **PVDF** | **NMP** | **Resulted membrane** | **Electrochemical measurements** |
| --- | --- | --- | --- | --- | --- |
| [OMIM][PF_6_] | 1 | 1 | 3 | Solid and homogeneous membranes were obtained, but they melted at room temperature. | - |
|  | 9 | 1 | 10 | No solid membranes were obtained. | - |
|  | 1 | 9 | 10 | The membrane could not be fixed on the surface of the devices and ended up falling to the solution. | - |
|  | 1.5 | 1 | 2.5 | Solid and homogeneous membranes were obtained. | No electrochemical signals were recorded due to the target analytes oxidation. |
|  | 1 | 1.5 | 2.5 |  |  |
|  | 1 | 1 | 5 |  |  |
|  | 1 | 2 | 3 |  |  |
|  | 2 | 2 | 3 |  |  |
|  | 1.5 | 1 | 3 |  |  |
|  | 2 | 1 | 3 | Solid and homogeneous membranes were obtained. | Although ethanethiol oxidation signals were electrochemical recorded, the corresponding to 4-ethylphenol wasn’t obtained. |
| [BMIM][PF_6_] | 1.5 | 1 | 3 | Solid and homogeneous membranes were obtained. | No electrochemical oxidation currents were recorded. |
|  | 2 | 1 | 3 | Solid and homogeneous membranes were obtained. | Although ethanethiol oxidation signals were electrochemical recorded, the corresponding to 4-ethylphenol wasn’t obtained. |
|  | 1 | 1 | 3 | Solid and homogeneous membranes were obtained. | Analogous oxidation currents were registered by the two electrodes. |
|  | 1.5 | 1.5 | 3 | Solid and homogeneous membranes were obtained. | Electrochemical signals were very noisy and not stable. |


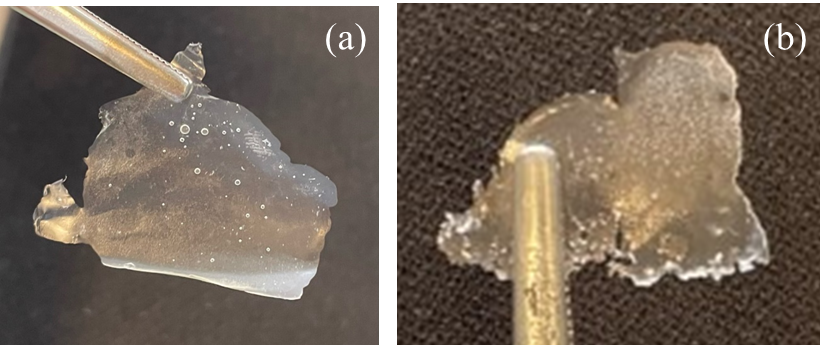


***Figure S2.-*** [OMIM]PF_6_ membranes obtained by mixing (A) 1:1:3 and (B) 1.5:1:2.5 of RTIL, PVDF and NMP and left to dry at 100 °C during 60 min.


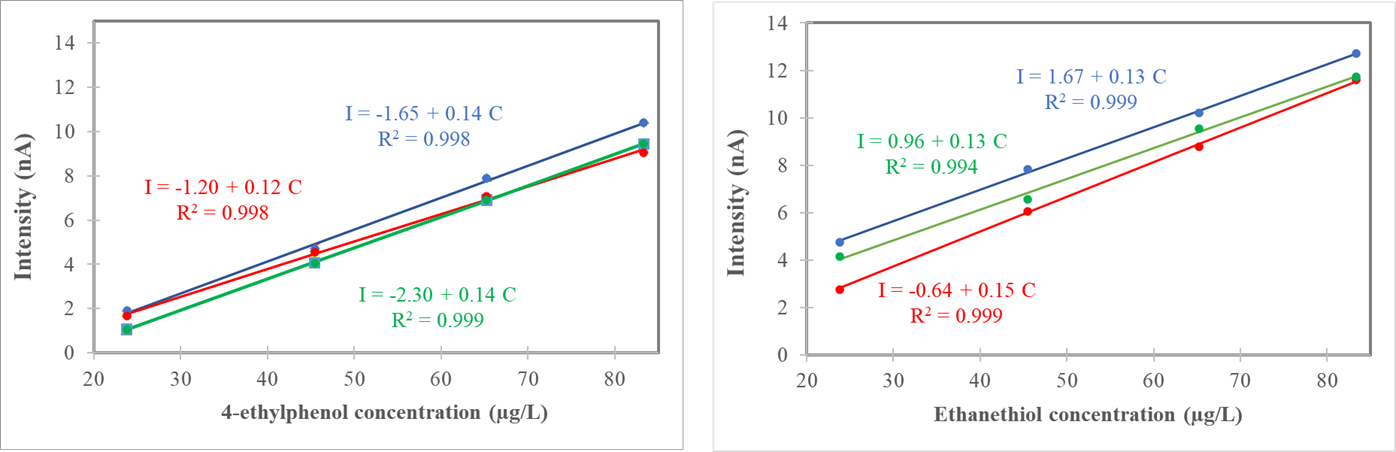


***Figure S3****. Experimental points and calibration plots obtained under optimized experimental conditions for the detection of 4-ethylphenol and ethanethiol using different AC_60_ and CoPh modified dual SPCEs coated with a [OMIM]PF_6_ based gel polymer electrolyte.*

***Table S2.-*** *Calibration parameters obtained through ordinary least squares regressions without outliers for the determination of 4-ethylphenol and ethanethiol using a single AC_60_ and CoPh modified dual SPCEs coated with a [OMIM]PF_6_ based gel polymer electrolyte under the optimum conditions.*

| **4-Ethylphenol concentration range (μg/L)** | **Intercept [nA]** | **Slope [nA (μg/L)^-1^]** | **Coefficient of determination, (R^2^)** | **Standard error of estimate (S_yx_)** |
| --- | --- | --- | --- | --- |
| 23.8 - 83.3 | -1.65 | 0.14 | 0.997 | 0.194 |
| 23.8 - 83.3 | -2.65 | 0.19 | 0.999 | 0.072 |
| 23.8 - 83.3 | -0.48 | 0.06 | 0.999 | 0.033 |
| **Ethanethiol concentration range (μg/L)** | **Intercept [nA]** | **Slope [nA (μg/L)^-1^]** | **Coefficient of determination, (R^2^)** | **Standard error of estimate (S_yx_)** |
| 23.8 - 83.3 | 1.67 | 0.13 | 0.999 | 0.136 |
| 23.8 - 83.3 | -0.12 | 0.10 | 0.990 | 0.341 |
| 23.8 - 83.3 | 2.16 | 0.01 | 0.947 | 0.039 |


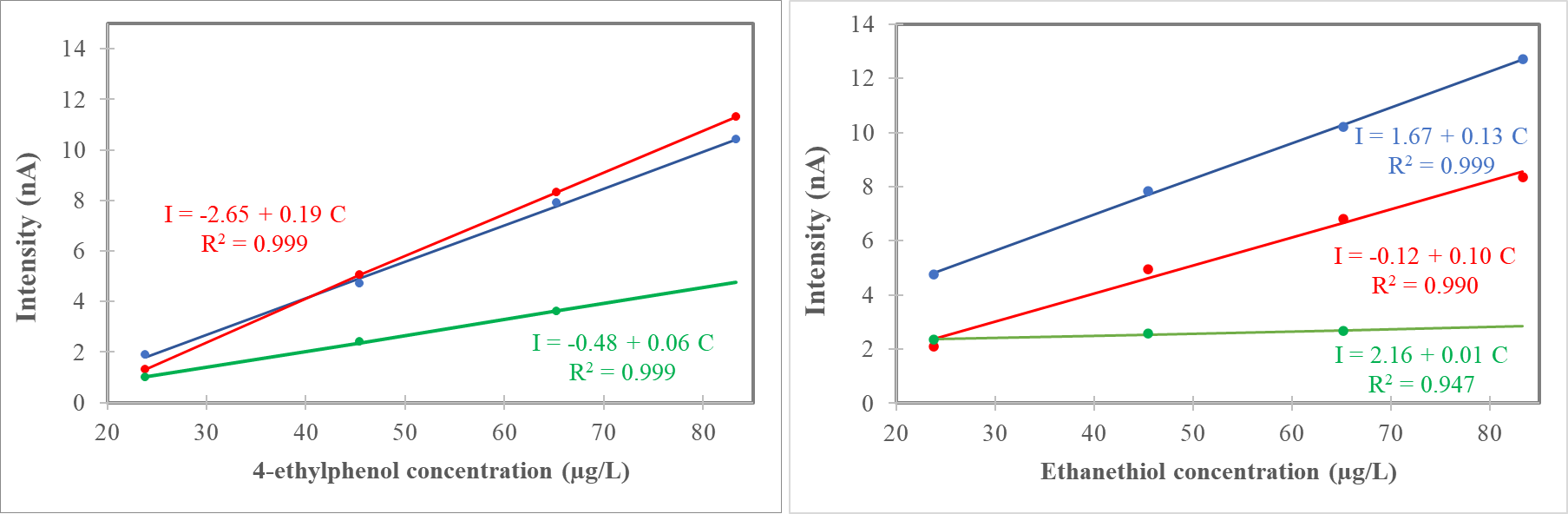


***Figure S4****. Experimental points and calibration plots obtained under optimized experimental conditions for the detection of 4-ethylphenol and ethanethiol using a single AC_60_ and CoPh modified dual SPCE coated with a [OMIM]PF_6_ based gel polymer electrolyte.*

***Table S3.-*** *Performance of gas electrochemical sensors for 4-ethylphenol and ethanethiol determinations in wine.*

| **Working electrode** | **Supporting electrolyte** | **4-Ethylphenol concentration range (μg/L)** | **Ethanethiol concentration range (μg/L)** | **Reproducibility** | **Capability of detection, α = β = 0.05** | **Limit of decision** | **Reference** |
| --- | --- | --- | --- | --- | --- | --- | --- |
| AC_60_-SPCE | 100 μL of BR buffer pH 5 and KCl placed onto the device after incubation for 6 min in gas phase | 700 - 1300 | Not detected | 5.4 % (n = 3) | 700 μg/L | Not shown | [21] |
| CoPh-SPCE | BR buffer pH 2.6 and KCl preloaded by adsorption | Not detected | 9.9 – 82.6 | 7 % (n = 3) | 12.5 μg/L | 6.5 μg/L | [23] |
| AC_60_ and CoPh modified dual SPCEs | [OMIM]PF_6_ based gel polymer electrolyte | 23.8 - 83.3 | 23.8 - 83.3 | 7.6 % (n = 3) for 4-ethylphenol and 6.6 % (n = 3) for ethanethiol | 23.8 μg/L for 4-ethylphenol and ethanethiol | 1.3 μg/L for 4-ethylphenol and 9.2 μg/L for ethanethiol | This work |
